# Preclinical Antiviral and Safety Profiling of the HBV RNA Destabilizer AB-161

**DOI:** 10.3390/v16030323

**Published:** 2024-02-21

**Authors:** Angela M. Lam, Ravi R. Dugyala, Muhammed Sheraz, Fei Liu, Emily P. Thi, Ingrid E. Graves, Andrea Cuconati, Holly Micolochick Steuer, Andrzej Ardzinski, Nathan Overholt, Jeremy D. Mason, Dimitar Gotchev, Andrew G. Cole, Troy O. Harasym, Michael J. Sofia

**Affiliations:** Arbutus Biopharma, Inc., 701 Veterans Circle, Warminster, PA 18974, USA; rdugyala@arbutusbio.com (R.R.D.); msheraz@arbutusbio.com (M.S.); fliu@arbutusbio.com (F.L.); ethi@arbutusbio.com (E.P.T.); igraves@arbutusbio.com (I.E.G.); acuconati@arbutusbio.com (A.C.); hsteuer@arbutusbio.com (H.M.S.); aardzinski@arbutusbio.com (A.A.); noverholt@arbutusbio.com (N.O.); jmason@arbutusbio.com (J.D.M.); dgotchev@arbutusbio.com (D.G.); acole@arbutusbio.com (A.G.C.); tharasym@arbutusbio.com (T.O.H.)

**Keywords:** HBV RNA, HBV RNA destabilizer, PAPD5, PAPD7

## Abstract

HBV RNA destabilizers are a class of small-molecule compounds that target the noncanonical poly(A) RNA polymerases PAPD5 and PAPD7, resulting in HBV RNA degradation and the suppression of viral proteins including the hepatitis B surface antigen (HBsAg). AB-161 is a next-generation HBV RNA destabilizer with potent antiviral activity, inhibiting HBsAg expressed from cccDNA and integrated HBV DNA in HBV cell-based models. AB-161 exhibits broad HBV genotype coverage, maintains activity against variants resistant to nucleoside analogs, and shows additive effects on HBV replication when combined with other classes of HBV inhibitors. In AAV-HBV-transduced mice, the dose-dependent reduction of HBsAg correlated with concentrations of AB-161 in the liver reaching above its effective concentration mediating 90% inhibition (EC_90_), compared to concentrations in plasma which were substantially below its EC_90_, indicating that high liver exposure drives antiviral activities. In preclinical 13-week safety studies, minor non-adverse delays in sensory nerve conductance velocity were noted in the high-dose groups in rats and dogs. However, all nerve conduction metrics remained within physiologically normal ranges, with no neurobehavioral or histopathological findings. Despite the improved neurotoxicity profile, microscopic findings associated with male reproductive toxicity were detected in dogs, which subsequently led to the discontinuation of AB-161’s clinical development.

## 1. Introduction

Globally, an estimated 257 million individuals are chronically infected with hepatitis B virus (HBV), which can progress to liver cirrhosis and hepatocellular carcinoma (HCC) [1]. Current approved therapies using pegylated interferon-α (PEG-IFNα) and nucleos/tide analogs (NA) result in very low rates of functional cure, which is defined as sustained HBV DNA suppression and hepatitis B surface antigen (HBsAg) loss with or without HBsAg seroconversion [2,3,4,5]. PEG-IFNα is associated with undesirable side effects, while lifelong therapy with NA is usually required to suppress viral replication. Data indicate that HBV viremia persists even with stable NA use, which may place the host at risk of liver-related morbidity and mortality [6,7].

The low rate of HBV functional cure is due, in part, to the persistence of covalently closed circular DNA (cccDNA), which serves as the transcriptional template for pregenomic RNA (pgRNA) and subgenomic messenger RNAs. These viral transcripts produce essential proteins to facilitate the replication and production of HBsAg-containing subviral particles (empty/non-infectious) and HBsAg-enveloped infectious virions. HBV can also be integrated into the host chromosome as a partial viral genome, which can be transcribed into subgenomic RNA and is another source of HBsAg. High levels of HBsAg are believed to contribute to host antiviral immune exhaustion, resulting in inadequate T cell and B cell responses [8,9]. Thus, sustained reduction of HBsAg may be an important step toward a functional cure. Reduction of HBsAg as a therapeutic strategy has been explored in recent clinical trials using injectable HBV-targeting *N*-acetylgalactosamine (GalNAc)-conjugated small interfering RNA (siRNA) and antisense oligonucleotide (ASO) to degrade HBV RNA [10,11,12,13]. An oral alternative to reduce HBsAg with a differentiated mechanism of action may be attractive for convenience of dosing administration and as a part of future combination therapies that may potentially improve the rate of functional cure. 

HBV RNA destabilizers are a class of small-molecule compounds with antiviral effects that are distinct from other direct-acting antivirals including NA and capsid assembly modulators (CAM). While NA and CAM primarily inhibit HBV DNA replication, HBV RNA destabilizers promote the degradation of viral transcripts by targeting the non-canonical poly(A) RNA polymerase-associated domain containing proteins 5 and 7 (PAPD5 and PAPD7). Mechanistic studies showed that PAPD5/7 form part of a protein-complex within the virus–host interaction pathway that HBV utilizes to stabilize its transcripts [14,15]. PAPD5/7-associated complex is recruited by the highly conserved stem loop-alpha (SLα) sequence located within the HBV post-transcriptional regulatory element (PRE). Upon recruitment onto the HBV RNA, PAPD5/7 facilitate extension of HBV RNA poly(A) tails with intermittent guanosine (G) [14,15]. The incorporation of intermittent G shields the poly(A) tails from de-adenylation, resulting in stabilization of HBV RNA [14]. Small molecules targeting PAPD5/7 lead to reduction of HBV pgRNA and HBsAg mRNA, thus representing a new antiviral strategy [15,16,17,18]. 

Among first-generation HBV RNA destabilizers, preclinical proof of concept has been achieved across a number of chronic infection animal models, including AAV-HBV-transduced mice [15,18,19], HBV-infected humanized liver chimeric mice [17,20], and woodchuck hepatitis virus (WHV)-infected woodchucks [21]. Multiple HBV RNA destabilizers have progressed into late-stage preclinical development (RG7834, AB-452, GS-8873), and some compounds were assessed in Phase 1 clinical studies including GST-HG131 [22], EDP-721 (NCT04971512), and GSK3965193 (NCT05330455). A number of these compounds have been discontinued due to undisclosed safety concerns during Phase 1a clinical studies (EDP-721) or observations of neurotoxicity during long-term preclinical tolerability studies (RG7834, GS-8873) [23,24,25]. Peripheral neurotoxicity (splayed limbs or decreased activity starting at 15 mg per kg) was also observed for AB-452, our first-generation HBV RNA destabilizer, during a 13-week repeat-dose safety study in dogs, which ultimately led to termination of AB-452 [25].

To overcome the hurdle of neurotoxicity, our effort focused on identifying a small-molecule compound with higher liver concentration and lower plasma exposures compared to AB-452. AB-161 was selected as a next-generation HBV RNA destabilizer based on its in vitro and in vivo antiviral potencies, favorable liver distribution in preclinical animal studies, and high plasma protein binding, which could reduce free compound concentration to limit systemic exposure and potentially reduce the risk of adverse events. Despite having a substantially improved neurological safety profile, adverse findings related to male reproductive toxicity were observed upon repeat dosing in dogs, which subsequently led to our decision to discontinue the development of AB-161. Herein, we report the preclinical antiviral characterization and safety tolerability observations from our studies of the second-generation HBV RNA destabilizer AB-161.

## 2. Materials and Methods

### 2.1. Compounds and Cell Culture

AB-161 and AB-836 were synthesized by Arbutus Biopharma. GLS-4, entecavir, and tenofovir disoproxil fumarate were purchased from Selleckchem (Houston, TX, USA).

HepG2.2.15 [26] (obtained from the Baruch S. Blumberg Institute, Doylestown, PA, USA), HepAD38 [27] (obtained from Christoph Seeger at the Institute for Cancer Research of Fox Chase Cancer Center, Philadelphia, PA, USA), and Huh-7 cells [28] (JCRB0403, Japanese Collection of Research Bioresources, Osaka, Japan) were maintained in DMEM/F12 medium supplemented with 1% penicillin–streptomycin (Pen/Strep), 10% fetal bovine serum (FBS), and 20 µg/mL G418 (for HepG2.2.15 and HepAD38). Human serum from non-HBV vaccinated donors was purchased from BioIVT (Westbury, NY, USA). PLC/PRF/5 cells [29], a human liver cancer cell line which synthesizes HBsAg from integrated HBV genome, were obtained from ATCC (American Type Culture Collection, Manassas, VA, USA). HepG2-hNTCP-C4 cells, a human hepatoma-derived cell line stably transfected with NTCP (Na^+^-taurocholate cotransporting polypeptide) [30], were obtained from the National Institute of Infectious Diseases (Tokyo, Japan) and were maintained in DMEM medium containing 10% FBS, Pen/Strep/L-glutamine, HEPES, 5 μg/mL insulin, and 400 µg/mL G418. Cryopreserved primary human hepatocytes (BioIVT) were maintained in Williams Medium E containing 0.1 μM dexamethasone and hepatocyte maintenance supplements (ThermoFisher Scientific, Waltham, MA, USA).

### 2.2. Antiviral Studies

HepG2.2.15 and PLC/PRF/5 cells were seeded in 96-well collagen-treated plates (3 × 10^4^ cells/well). After overnight incubation, cells were treated with compounds serially diluted in DMSO, and compounds were replenished three days later. Following a 6 day-incubation, media were removed for HBsAg and HBeAg chemiluminescence immunoassay (CLIA) according to the manufacturer’s recommendation (AutoBio Diagnostics, Zhengzhou, China). Secreted HBV DNA was extracted (Realtime Ready Cell Lysis Kit, Roche, Mannheim, Germany) and quantified by PCR using HBV specific primers (5′-GGC TTT CGG AAA ATT CCT ATG and 5′-AGC CCT ACG AAC CAC TGA AC) using the conditions of denaturing at 95 °C for 5 min, followed by 40 cycles of amplification at 95 °C for 15 s and 60 °C for 30 s. Antiviral selectivity studies against a panel of different DNA and RNA viruses were performed at ImQuest Biosciences (Frederick, MD, USA). To determine cytotoxic effect of the compounds, cells were seeded at subconfluent density (5000 cells/well) and treated with compounds as described above. Cell viability was determined by measuring intracellular adenosine triphosphate (ATP) using CellTiter-Glo (Promega, Madison, WI, USA). For combination studies, HepG2.2.15 cells were treated with AB-161 with or without ETV, TDF, or AB-836 at a range of concentrations in a checkerboard format. Secreted HBV DNA and HBsAg levels were measured, and results were analyzed using the method of Prichard and Shipman [31]. 

### 2.3. Infection of HepG2-hNTCP Cells and Primary Human Hepatocytes

HepG2-hNTCP-C4 cells were seeded into collagen-coated 10 cm dishes (8.6 × 10^6^ cells/dish) and cultured in complete DMEM medium containing 2% DMSO. One day later, cells were infected with HBV inoculum (collected and purified from supernatants of HepAD38 cells by Imquest Biosciences, Frederick, MD) at 250 genome equivalent (GE)/cell in DMEM containing 4% PEG-8000. The inoculums were removed 24 h later and the infected cells were washed 4 times with PBS and seeded in 96-well plates (5 × 10^4^ cells/well). Serially diluted compounds were added, and cells were incubated for 5 days with compounds. For the infection of primary human hepatocytes (PHHs), cells were seeded in collagen-coated 96-well plates (65,000 cells/well) overnight then infected with HBV at 250 GE/cell in media containing 4% PEG-8000. The inoculums were removed 24 h later and compounds were added or replenished on days 4, 7, 9, 11, and 14 post-infection. On day 16, supernatants were collected for HBsAg and HBeAg quantification as described above. To quantify HBV RNA, total cellular RNA was extracted from infected cells using RNeasy 96 kit per the manufacturer’s recommendation (Qiagen, Hilden, Germany). HBV RNA and GAPDH housekeeping transcripts were amplified using HBV primers and probe (5′-GTC CTC AAY TTG TCC TGG, 5′-TGA GGC ATA GCA GCA GGA T, /56-FAM/CTG GAT GTG TCT GCG GCG TTT TAT CAT/36-TAMSp/) (Integrated DNA Technologies) and GAPDH primers and probe (ThermoFisher Scientific), respectively, and quantified by TaqMan™ Fast Virus 1-step Master Mix on a QuantStudio™ 7 Flex Real-Time PCR System (ThermoFisher Scientific). Reductions in RNA values were determined by calculating the ΔCt values from a non-treated infected control. Dose–response curves and the 50% effective concentration (EC_50_) were generated using GraphPad Prism’s non-linear regression response variable slope function (4 parameters). 

### 2.4. HBV Genotype Coverage and HBV Variants Phenotyping Studies

Huh-7 cells seeded in 100 mm dishes (4.5 × 10^6^) were transfected with HBV plasmids harboring a 1.1-mer-overlength genotype D HBV (pcDNA3.1-HBV1.1) consisting of genotype A (GenBank AF537372), B (GenBank KU964112), C (GenBank KU964186), D (GenBank U95551), E (GenBank HE974380), F (GenBank AB036920), H-1 (GenBank AB064315), and H-2 (GenBank AB516394). Huh-7 cells were also transfected with HBV plasmids containing amino acid changes within the HBV reverse transcriptase (L180M/M204V and L180M/M204V/T184G/S202I), which were introduced into the genotype D HBV plasmid using site-directed mutagenesis. Briefly, plasmid DNA was mixed with Lipofectamine™ 3000 in Opti-MEM^®^ I Reduced-Serum Medium (ThermoFisher Scientific), incubated at room temperature for 15 min, then transferred to Huh-7 cells. After about 6 h incubation at 37 °C with the transfection mixture, cells were trypsinized and plated into 96-well plates (3 × 10^4^ cells/well) containing serially diluted compounds. Two days later, the medium was removed and replenished for another 3 days. Culture supernatants were collected for HBsAg quantification using the CLIA assay as described above.

For the HBV PRE variant phenotyping studies, Huh-7 cells were transfected with plasmids containing the HBsAg encoding gene followed by the full-length wildtype HBV PRE sequence or the SLα deletion mutant (ΔSLα, nt 1294 to 1322) (GenScript, Piscataway, NJ, USA). Expression of HBsAg was under the regulation of tetracycline-controlled CMV promoter. Cells were treated with or without AB-161 for 5 days, after which culture supernatants were collected for HBsAg quantitation. 

### 2.5. Northern Blot Analysis of Intracellular HBV RNA

Total cellular RNA from HepG2.2.15 cells or HepAD38 cells was extracted using TRIzol™ reagent (ThermoFisher Scientific) according to the manufacturer’s direction. Total RNA (6 µg) was fractionated in a 1.5% agarose gel containing 2.2 M formaldehyde and transferred onto a Hybond-XL™ membrane in 20× saline sodium citrate (SSC) buffer. To detect HBV RNA, membranes were probed with a digoxigenin (DIG)-labeled minus-strand specific full-length HBV riboprobe transcribed from plasmid pSP65_HBV DNA. Membranes were hybridized with DIG-specific antibody, conjugated with alkaline phosphatase enzyme. Membranes were exposed to SuperSignal™ West Pico PLUS Chemiluminescent Substrate and quantified using iBright™ FL100 Imaging System (ThermoFisher Scientific).

### 2.6. Particle Gel Analysis of Nucleocapsid and Southern Blot Analysis of Intracellular HBV DNA

For intracellular viral nucleocapsid analysis, HepG2.2.15 cells treated with HBV inhibitors were lysed in buffer containing 10 mM Tris-HCl (pH 7.6), 100 mM NaCl, 1 mM EDTA, and 0.1% NP-40. Viral particles were fractionated through nondenaturing 1% agarose gels electrophoresis and transferred to a nitrocellulose filter by blotting with TNE buffer (10 mM Tris-HCl [pH 7.6], 150 mM NaCl, and 1 mM EDTA). To detect HBV core antigens, membranes were probed with polyclonal antibody against HBV core protein (Dako, Glostrup, Denmark). Bound antibodies were revealed by horseradish peroxidase-labeled secondary antibodies and visualized with the iBright™ Imaging Systems (ThermoFisher Scientific). For the detection of encapsidated HBV DNA, the DNA-containing particles on the membrane were denatured with a solution containing 0.5 M NaOH and 1.5 M NaCl, and this step was followed by neutralization with a solution containing 1 M Tris-HCl (pH 7.6) and 1.5 M NaCl. HBV DNA was detected via hybridization with an α-^32^P-UTP-labeled full-length HBV riboprobe complimentary to the minus-strand of viral DNA.

### 2.7. Western Blot Analysis of HBV Core

HepG2.2.15 cells treated with HBV inhibitors were lysed with Laemmli sample buffer (Bio-Rad, Philadelphia, PA, USA) supplemented with 2.5% 2-mercaptoethanol (Sigma-Aldrich, St. Louis, MO, USA). Cell lysates were fractionated by denaturing gel electrophoresis with 12% Criterion TGX Stain-Free Precast Gels and Tris/Glycine/SDS running buffer (Bio-Rad). Proteins were transferred onto a polyvinylidene difluoride (PVDF) membrane, blocked with 5% nonfat milk in TBS-0.1% Tween for 1 h, and incubated with the anti-HBcAg antibody (Dako) or anti-beta actin antibody (Abcam, Cambridge, UK) at 4 °C overnight. After washing with TBST (TBS containing 0.1% Tween 20), the membrane was incubated with the secondary antibody. HBV core was detected using Clarity™ Western ECL Substrate (Bio-Rad) and imaged with the iBright™ Imaging Systems (ThermoFisher Scientific). 

### 2.8. PAPD5 and PAPD7 ATP Depletion Assay

Cloning, expression, and purification of recombinant human PAPD5 (amino acids 186-518, NCBI Reference Sequence: XM_011523275) and PAPD7 (amino acids 226-558, NCBI Reference Sequence: NM_006999.6) were previously described [15]. Briefly, AB-161 was added to recombinant PAPD5 or PAPD7 proteins (12.5 nM) in 10 mM Tris-HCl (pH 8.0), 100 mM KCl, 5 mM MgCl_2_, 250 nM RNA substrate (5′-GCC UUU CAU CUC UAA CUG CGA AAA AAA AAA), 750 nM ATP, 0.1 mM EDTA, 1 mM TCEP, and 0.002% NP-40. The reactions were incubated at room temperature for 3 h and ATP depletion was monitored by using Kinase-Glo^®^ Luminescent Kinase kit following manufacturer’s instructions (Promega, Madison, WI, USA).

### 2.9. In Vivo Antiviral Activity and Pharmacokinetics Analysis Using AAV-HBV Mouse Model

Male C57BL/6J mice, 6 weeks old (The Jackson Laboratories, Sacramento, CA, USA), were each inoculated with 1 × 10^11^ genomes of adeno-associated virus (AAV) vector AAV-HBV containing a 1.2× overlength sequence of HBV genome (genotype D, GenBank accession no. V01460) (SignaGen Laboratories, Rockville, MD, USA). Mice were administered the AAV2/8-type vector via intravenous tail vein injection. Twenty-eight days after AAV infection, animals were randomized into groups (*n* = 6 or 9) based on serum HBsAg concentration. Animals were administered vehicle only or AB-161 at 0.3, 1, 10, or 30 mg/kg via oral gavage once daily for 14 days and terminated 24 h after the last dose. Serum was collected on study days −1, 5, 8, 11, and 15, and HBsAg concentrations were determined using the GS HBsAg EIA 3.0 kit (Bio-Rad, Redmond, WA, USA) according to the manufacturer’s protocol against a 2-fold serially diluted recombinant HBsAg protein standard (Abcam, Waltham, MA, USA) and read on a SpectraMax^®^ Plus plate reader (Molecular Devices, San Jose, CA, USA). To measure the concentrations of AB-161 in plasma and in liver, plasma samples were precipitated with acetonitrile containing an internal standard, while livers were homogenized in 1:4 volumes of PBS, centrifuged for 5 min at 4000 rpm to obtain the supernatant, and precipitated similarly as with the plasma samples. Standard curves of AB-161 were prepared by using both plasma and liver homogenates. Compound concentrations in both matrices were determined by LC-MS/MS on a SCIEX QTrap 5500. Animal-related procedures were conducted by Inotiv personnel at St. Louis University Medical School’s Department of Comparative Medicine, an Association for Assessment and Accreditation of Laboratory Animal Care International (AAALAC International)-certified facility registered with the US Department of Agriculture’s Animal and Plant Health Inspection Service (USDA-APHIS). All study protocols were reviewed by St. Louis University’s Institutional Animal Care and Use Committee (IACUC) prior to study initiation (IACUC approved animal use protocol number SLU-2481). During the study, the care and use of animals were conducted to comply with the Animal Welfare Act (9 CFR Parts 1, 2, and 3), follow the Guide for the Care and Use of Laboratory Animals (Eight Edition, Institute of Laboratory Animal Resources, National Academy Press, Washington, DC, USA, 2011).

### 2.10. Plasma Protein Binding Assay

Plasma protein binding of the test compound and positive controls (diclofenac sodium salt, letrozole, and fluconazole) were determined by using a 96-well HTD96B equilibrium dialysis device (Fisher Scientific, Hampton, NH) with a 12–14 kDa membrane (regenerated cellulose dialysis tubing, Fischer Scientific). Pooled mouse, rat, dog, monkey, and human plasma were purchased from BioIVT. Prior to the dialysis plate set-up, the membranes were hydrated in Dulbecco’s phosphate buffered saline (DPBS) for 60 min followed by a 20-min wash in 20% ethanol and rinsed twice with DPBS. All evaluations were performed in triplicate. AB-161 or control compounds were spiked into plasma for a final concentration of 500 ng/mL (1.15 µM). Spiked plasma (125 μL) was added to the donor side of the plate, and an equal volume of DPBS was added to the receiver side. After incubation for 6 h at 37 °C under 5% CO_2_ environment, samples were precipitated, and the supernatant was analyzed using a Waters Alliance 2795 LC and Quattro MS. The bound fraction (f_B_) was calculated using the formula: f_B_ = 1 − (PAR_Receiver_/PAR_Donor_), where PAR_Receiver_ = mean peak area ratio from receiver side; PAR_Donor_ = mean peak area ratio from the donor side.

### 2.11. Repeat Dose Toxicity Studies

#### 2.11.1. Animals

Non-clinical 13-week repeat dose toxicity studies were performed using rats and dogs at Charles River Laboratories Ashland, LLC (Ashland, OH, USA), an AAALAC certified facility, in compliance with ICH Guidelines and in adherence with Good Laboratory Practice (GLP) requirements with few exceptions. Nerve conduction velocity (NCV) measurements were performed in accordance with study protocols and animal welfare guidelines. NCV data collection and reporting underwent a quality control review to ensure data integrity. Study protocols were reviewed and approved by the Charles River Ashland Institutional Animal Care and Use Committee before conduct (IACUC approved protocol numbers 01041051 and 01041052). During the study, the care and use of animals were conducted with guidance from the guidelines of the USA National Research Council Guide for the Care and Use of Laboratory animals and the Office of Laboratory Animal Welfare Public Health Services Policy on Human Care and Use of Laboratory Animals, NIH, Bethesda, MD, USA.

In the 13-week repeat-dose rat toxicity study, Sprague Dawley (SD) rats (Crl:CD) were received from Charles River Laboratories (Raleigh, NC, USA) and, at the time of dose initiation, were approximately 13–14 weeks old. Males weighed between 403 and 552 g and the females weighed between 233 and 326 g at the time of dosing. Animals were group housed and rooms were maintained at 68 °F to 78 °F with a humidity of 30% to 70% and a set 12 h light and 12 h dark cycle except during designated procedures. Food and water were available ad libitum throughout the in-life phase of the study.

In the 13-week repeat-dose dog toxicity study, Beagle dogs were obtained from Marshall Bioresources (North Rose, NY, USA) and, at the time of dose initiation, were approximately 6–7 months old. Males weighed between 6.5 and 8.5 kg and the females weighed between 5.4 and 7.6 kg. Animals were group housed and rooms were maintained at 66 °F to 76 °F with a humidity of 30% to 70% and a set 12 h light and 12 h dark cycle except during designated procedures. Food and water were available ad libitum throughout the in-life phase of the study.

#### 2.11.2. Study Design and Nerve Conduction Assessments

In the 13-week rat repeat-dose toxicity study, 10 animals/sex/group were administered AB-161 at doses of 0, 30, 120, and 500 mg/kg/day for 91–98 days via oral gavage. Additionally, 5 animals/sex in the control group and the 500 mg/kg/day group were also assigned for a 28-day recovery period. In the 13-week dog repeat-dose toxicity study, 4 animals/sex/group were administered AB-161 at doses of 0, 10, 30, and 100 mg/kg/day for at least 91 days via oral gavage. Additionally, 2 animals/sex in the control group and 100 mg/kg/day group were assigned for a 28-day recovery period. Vehicle control for both studies was 0.5% (*w*/*v*) methylcellulose 400, 0.1% (*v*/*v*) Tween 80 in 50 mM sodium bicarbonate buffer pH 9 ± 0.1, and the dose volume was 10 mL/kg. The following parameters and end points were evaluated: mortality, clinical signs, body weights, food consumption, ophthalmology, electrocardiography (dog study only), nerve conduction assessments, neurobehavioral assessments, motor activity, clinical pathology parameters (hematology, coagulation, clinical chemistry, and urinalysis), toxicokinetic parameters, organ weights, and macroscopic and microscopic examinations.

Nerve conduction assessments were conducted during pretreatment, on dosing period day 68–70 (rats) and 71–72 (dogs), and at the end of recovery period on day 119. After anesthesia, nerve conduction was assessed by a certified Natus Neurology System (rats) and a clinical electromyography machine (dogs). Subdermal needle electrodes were used for both stimulation and recording. Evaluations in rats were conducted on the caudal nerve (both sensory and motor), the sciatic/tibial nerve (motor), or peroneal nerve (sensory). In dogs, nerve conduction function was assessed in one sensory nerve of the lower limb and one motor nerve innervating distal muscles (sciatic/tibial or peroneal nerve). Conduction velocities were calculated via preset software protocols using the onset latency of the response and the distance to the cathode. Statistical comparisons were conducted at each timepoint and across timepoints.

Data were presented as means, standard deviations, ratio, and percentages, and numbers and/or incidences were reported as appropriate by dataset. For the organ weight data, groups were compared using an overall one-way ANOVA F-test, and if found to be significant, then pairwise comparisons were conducted using Dunnett’s test. Statistical analysis was not conducted in the recovery groups because of small group size (*n* = 2/group). For the NCV assessment, statistical analyses for action potential metrics included a Levene’s test to assess the homogeneity of group variances and an analysis of variance (2-sided ANOVA) that included the treatment group as the fixed effect (by time point and sex). Where the overall F-test was significant, each treated group was compared to control using Dunnett’s test. In cases where the Levene’s test was significant, a Krustal–Wallis test was performed instead of the ANOVA. All statistical analyses were performed at a 5% significant level. 

## 3. Results

### 3.1. AB-161 Selectively Inhibits HBsAg, HBeAg, and HBV DNA In Vitro

**Figure 1:**
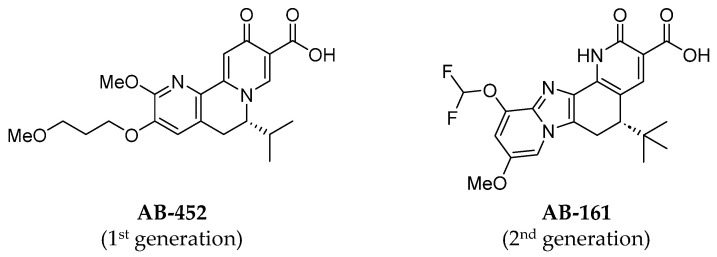
Chemical structures of AB-452 and AB-161.

AB-161 incorporates a novel tetracyclic 2-pyridone chemical modification and is structurally differentiated from the first-generation compound AB-452 and other known dihydroquinolizinones (DHQ) compounds (Figure 1). To determine its antiviral potency, AB-161 was evaluated using multiple in vitro HBV cell-based systems (Table 1). In HepG2.2.15 cells [26], AB-161 reduced HBsAg in a concentration-dependent manner with mean EC_50_ of 2.3 nM. AB-161 also reduced the levels of secreted HBeAg and HBV DNA with EC_50_ values of 13.7 nM and 0.36 nM, respectively. No apparent cytotoxicity was observed up to the highest concentration tested (CC_50_ > 30 µM). To examine whether the presence of human serum may attenuate compound efficacy, the potency of AB-161 was measured in HepG2.2.15 cells with or without 40% human serum. Results showed an approximately 30-fold increase in the EC_50_ value for AB-161 against HBsAg in HepG2.2.15 cells (Table 1).

Primary human hepatocytes (PHH) and HepG2 cells expressing the human NTCP (HepG2-hNTCP) are both cccDNA-dependent HBV replication models and represent a more biologically relevant system. In HBV-infected PHH, AB-161 inhibited HBsAg with EC_50_ of 8.5 nM. AB-161 also inhibited HBeAg and HBV RNA with EC_50_ values of 11.9 nM and 9.6 nM, respectively. In HBV-infected HepG2-hNTCP cells, AB-161 inhibited HBsAg, HBeAg, and HBV RNA with EC_50_ values of 25.3 nM, 20.9 nM, and 30.1 nM, respectively. Furthermore, the antiviral effect of AB-161 against HBsAg produced from integrated HBV was examined using the PLC/PRF/5 cell line, which contains partially integrated HBV genome [32]. Results showed that AB-161 efficiently inhibited the production of HBsAg expressed from integrated HBV (EC_50_ = 5.9 nM).

To evaluate the HBV genotype coverage of AB-161, Huh-7 cells transiently transfected with plasmid DNA harboring representative HBV isolates from genotypes A, B, C, D, E, F, H-1, and H-2 were treated with AB-161, and levels of HBsAg were monitored from cultured supernatants. AB-161 efficiently suppressed HBsAg from cells transfected with the representative HBV isolates, with EC_50_ values that ranged from 2.1 to 4.5 nM (Table 1 and Table S1). These data suggest that AB-161 would have broad HBV genotype coverage.

The selectivity of AB-161 was examined by evaluating its activity against a panel of RNA and DNA viruses. Results showed that AB-161 was inactive against all ten viruses up to the highest concentration tested at 30 μM, suggesting that AB-161 is highly selective for HBV (selectivity > 10,000-fold) (Table S2).

### 3.2. Nucleos/Tide Resistant Variants Remained Fully Susceptible to AB-161

Chronic hepatitis B (CHB) patients undergoing long-term NA therapy may develop resistance leading to treatment failure. To evaluate the activity of AB-161 against HBV polymerase variants known to confer resistance to certain NA, HBV plasmids encoding either wild-type or HBV reverse transcriptase (rt) variants (rtL180M/M204V and rtL180M/M204V/T184G/S202I) were transfected into Huh-7 cells and subsequently treated with AB-161. The NA entecavir (ETV) and the HBV CAM GLS4 were included as positive and negative controls, respectively. AB-161 showed strong antiviral activities against these NA-resistant variants, with similar EC_50_ values as wild-type HBV (EC_50_ = 1.9 to 4.9 nM) (Table 2). On the contrary, HBV DNA replication in cells transfected with the NA-resistant variants were less sensitive to ETV when compared to those transfected with wild-type HBV (EC_50_ increased from 16- to >370-fold). As expected, these NA-resistant variants remained susceptible to inhibition by GLS4 (Table 2). These data suggest that AB-161 retains antiviral activity against HBV variants that confer resistance to NA analogs. 

### 3.3. Combination of AB-161 with NA or CAM Shows Additive Antiviral Effects

The goal of novel CHB therapies is to increase the functional cure rate with a finite treatment duration. The next wave of HBV antivirals being developed may be combined with standard of care or investigational agents with complimentary mechanisms to enhance viral suppression. To assess the potential of combinations, in vitro studies were conducted in HepG2.2.15 cells to combine AB-161 with ETV, tenofovir disoproxil fumarate (TDF), or the investigational HBV CAM, AB-836. When each of the compounds was tested alone, all compounds showed potent inhibition of HBV DNA (Table 3). Combining AB-161 with ETV, TDF, or AB-836 resulted in additive effects against HBV DNA, with no antagonism observed (Table 3). While ETV, TDF and AB-836 are potent inhibitors against HBV replication, they do not affect the levels HBsAg. In this system, ETV, TDF, and AB-836 did not interfere with HBsAg inhibition when combined with AB-161. Furthermore, neither the individual nor the combination treatments resulted in significant loss of cell viability compared to untreated controls (≤20%) across all tested concentrations.

### 3.4. Inhibition of PAPD5 and PAPD7 by AB-161 Facilitates HBV RNA Destabilization

**Figure 2:**
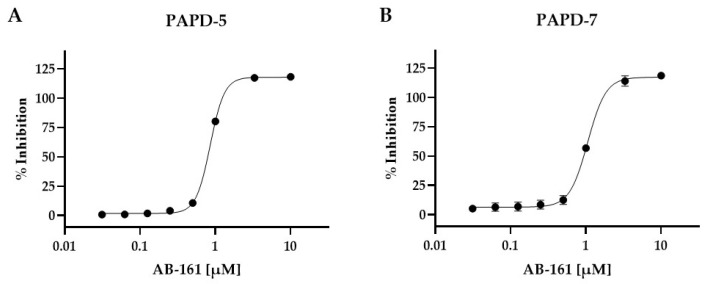
AB-161 inhibits PAPD5 and PAPD7 enzymatic activity. (**A**) Recombinant human PAPD5 or (**B**) PAPD7 was incubated with increasing concentrations of AB-161 and the inhibitory effect against ATP incorporation was measured. Half maximal inhibition (IC_50_) was determined based on the dose–response curves. Mean IC_50_ values were determined from 3 independent experiments.

The targets of HBV RNA destabilizers, exemplified by RG7834, were reported to be PAPD5 and PAPD7 [16,33]. We and others have also reported on the mechanism by which PAPD5/7 protected HBV RNA through stabilizing its poly(A) tails [14,15]. We determined the effect of AB-161 on the enzymatic activity of recombinant human PAPD5 and PAPD7 by monitoring the enzymes’ ability to incorporate ATP. Results showed that AB-161 inhibited PAPD5- and PAPD7-mediated incorporation of ATP into an RNA substrate with mean IC_50_ values of 0.85 and 1.1 µM, respectively (Figure 2). In addition, the stem-loop alpha (SLα) sequence within the HBV PRE has been demonstrated to play a role in recruiting PAPD5/7-containing complex onto HBV RNA, and that SLα was critical for the antiviral activity of HBV RNA destabilizers [15,18]. To determine whether the SLα sequence was also essential for AB-161 activity, Huh-7 cells were transfected with plasmids containing either wild-type HBV PRE (WT) or a deleted SLα (ΔSLα, nt 1295-1321) (Figure S1). Both plasmids contained the HBsAg open reading frame and the HBV poly(A) signal sequence. Results showed that AB-161 effectively suppressed HBsAg expression from Huh-7 cells transfected with the WT plasmid with an EC_50_ value of 2.5 nM. On the contrary, no apparent inhibition of HBsAg by AB-161 was observed in cells transfected with the ΔSLα plasmid (EC_50_ > 100 nM), suggesting that the HBV PRE region and specifically the SLα sequence was essential for AB-161 antiviral activity (Figure S1).

**Figure 3:**
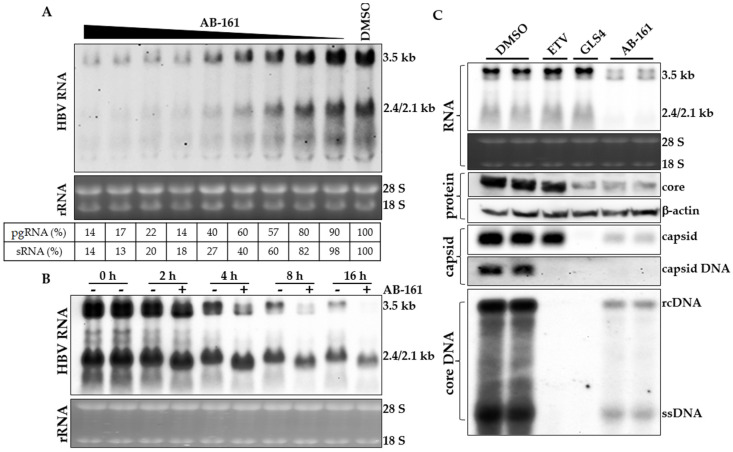
AB-161 destabilized intracellular HBV RNA and suppressed viral protein production and replication. (**A**) HepG2.2.15 cells were treated with DMSO or AB-161 at increasing concentrations (0.3 to 1700 nM) for 48 h. The relative intensities of HBV pgRNA (3.5 kb) and preS/S RNA (2.4/2.1 kb) in each sample are expressed as percentages of those from untreated cells and are indicated below each lane. (**B**) Time course analysis of HBV pgRNA and preS/S RNA from HepAD38 cells treated with GLS4 in the presence or absence of AB-161 (100 nM). Total RNA was extracted before the addition of AB-161 (0 h) or at 2, 4, 8, and 16 h post-addition of AB-161. Ribosomal 28S and 18S rRNA were included as loading controls. (**C**) Interference of HBV life cycle by AB-161. HepG2.2.15 cells were treated with DMSO, ETV (1 μM), GLS4 (1 μM), or AB-161 (100 nM). Intracellular HBV pgRNA and preS/S RNA were analyzed via Northern blot, HBV core via Western blot (β-actin as loading control), HBV capsid and encapsidated DNA via particle gel assay, HBV rcDNA and ssDNA via Southern blot.

Since AB-161 inhibited the enzymatic activities of PAPD5 and PAPD7, this mechanism of action was anticipated to destabilize HBV RNA. To determine the effect of AB-161 on intracellular HBV transcripts, total RNA was extracted from HepG2.2.15 cells and viral RNA was examined via Northern blot. Results showed that AB-161 reduced the steady state level of the pgRNA (3.5 kb) and the preS/S RNA (2.4/2.1 kb) with EC_50_ between 6.9 to 21 nM and 2.3 to 6.9 nM, respectively (Figure 3A). The concentration of AB-161 needed to reduce HBV RNAs correlated well with that of secreted HBsAg inhibition (Table 1). 

The kinetics of HBV RNA degradation promoted by AB-161 were next examined using HepAD38 cells in which HBV transcription is under tetracycline (Tet) regulation. HepAD38 cells were induced to initiate HBV transcription and were treated with the HBV CAM GLS4 to block encapsidation of pgRNA [34,35]. Six days later, Tet was added back to shut down transcription and cells were treated with both GLS4 and AB-161 for an additional 16 h. The decay of HBV pgRNA and preS/S RNA was monitored over time (Figure 3B). In the absence of AB-161, levels of pre-transcribed HBV RNA reduced with time due to natural turnover of viral transcripts. In the presence of AB-161, both pgRNA and preS/S RNA exhibited faster migration starting at 2 h post-treatment, indicating shortening of the viral transcripts. In addition, AB-161 substantially reduced the levels of both pgRNA and preS/S RNA at 8 h and 16 h post-treatment (Figure 3B). 

Since AB-161 not only reduced the level of preS/S RNA but also pgRNA, the data suggest that multiple stages of the HBV life cycle would be suppressed in the presence of AB-161. To evaluate the effect of AB-161 on the viral life cycle, intracellular HBV intermediates and viral proteins were analyzed from HepG2.2.15 cells treated with AB-161. ETV and GLS4 were included as reference compounds inhibiting viral polymerase and capsid assembly, respectively. While ETV profoundly inhibited HBV DNA replication, it had no effect against HBV RNA or viral proteins. As a CAM that induces the formation of aberrant capsids (CAM-A), GLS4 substantially reduced HBV core and capsid assembly, as well as DNA replication. However, GLS4 showed no effect against intracellular HBV RNA. On the contrary, AB-161 displayed a very distinct antiviral phenotype as it inhibited multiple stages of the HBV life cycle including HBV pgRNA, preS/S RNA, HBV core and capsids, and replicating HBV DNA (Figure 3C).

### 3.5. Reduction of HBsAg In Vivo Is Associated with AB-161 Concentration in Liver

The primary target organ for HBV is the liver, where viral replication, protein expression, and viral particle assembly occurs in infected hepatocytes. As demonstrated using in vitro HBV systems, AB-161 acts on HBV viral RNA transcripts including those encoding viral proteins HBsAg, HBeAg, polymerase, and HBcAg, as well as the genomic replication intermediate, pgRNA, which undergoes reverse-transcription to form rcDNA in newly assembled virions. To examine antiviral efficacy in vivo, AB-161 was evaluated in an AAV-HBV transduced mouse model as a surrogate of chronic HBV infection [36,37]. 

**Figure 4:**
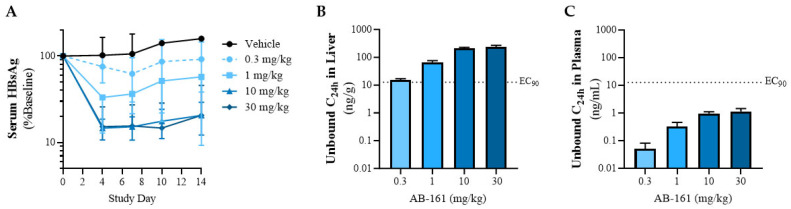
AB-161 mediates reduction in HBsAg in an AAV-HBV mouse model. (**A**) AAV-HBV mice received once-daily oral doses of AB-161 or vehicle control from Days 0 to 13. Data represent serum HBsAg levels relative to pre-dose baseline levels of each animal. HBsAg was assessed via ELISA. Data represent group mean (*n* = 6–9) ± standard deviation. (**B**) AB-161 unbound concentrations in liver and (**C**) plasma at 24 h post-last dose (C_24h_). Unbound concentrations were calculated by applying a fraction-unbound factor of 0.024 to total C_24h_ liver concentrations measured by LC-MS. Fraction-unbound factor represents 2.4% of AB-161, estimated to be free based on 97.6% mouse plasma protein binding.

Daily oral dosing (QD) of 0.3, 1, 10, or 30 mg/kg AB-161 for 14 days in AAV-HBV mice resulted in a mean maximal 0.94 ± 0.56 log_10_ reduction of serum HBsAg compared to pre-dose baseline levels. Inhibition of serum HBsAg was observed as early as 4 days after initiation of dosing and was maintained with repeat dosing. Dose-responsive declines in serum HBsAg were observed across the QD dose range tested of 0.3, 1, 10, and 30 mg/kg, which resulted in mean reductions of 0.13, 0.34, 0.71, and 0.94 log_10_ compared to pre-dose baseline levels, respectively (Figure 4A). 

As the activity of AB-161 is impacted by human serum in HepG2.2.15 cells (Table 1), this suggests that the presence of serum proteins likely reduces the levels of free compound through protein sequestering of AB-161. To determine the extent of protein binding, in vitro plasma protein binding studies were conducted using plasma from human, mouse, rat, dog, and monkey. Consistent with the cell-based data, AB-161 exhibited high plasma protein binding across multiple species, with 95.1%, 97.6%, 94.4%, 95.3%, and 98.0% of AB-161 remaining bound in plasma from human, mouse, rat, dog, and monkey, respectively. Considering the high plasma protein binding effect, the fraction-unbound concentrations of AB-161 from AAV-HBV mouse plasma and liver at 24 h post-last dose (C_24h_) were calculated and compared to the EC_90_ of AB-161 (EC_90_ = 12.8 ng/mL). Results show that reductions of HBsAg were observed when fraction-unbound concentrations of AB-161 in the liver were at or greater than the EC_90_ value (Figure 4B). On the contrary, the levels of fraction unbound AB-161 in plasma were at least 1 log_10_ below its EC_90_ across all doses tested (Figure 4C), suggesting that the antiviral efficacy observed in this AAV-HBV mouse model was driven by the high AB-161 concentrations in liver. 

AB-161 once-daily dosing for 14 days was well-tolerated in AAV-HBV mice, with no significant changes in body or liver weights and no significant changes in liver and kidney function markers (Figure S2). These results demonstrate that daily oral administration of AB-161 results in anti-HBV activity in vivo and is associated with higher liver concentrations when compared to levels measured in plasma.

### 3.6. 13-Week Repeat Dose Toxicity Studies in Rats and Dogs

Preclinical toxicology evaluation of first-generation HBV RNA destabilizers (GS-8873, RG7834) indicates that the long-term administration of these compounds led to adverse findings associated with neurotoxicity [23,24]. The clinical development of our first-generation HBV RNA destabilizer, AB-452, was also halted due to the observation of peripheral neuropathy in preclinical studies in dogs, during which uncoordinated movement or splayed limbs were observed [25]. As such, to evaluate the effects of long-term repeat dosing of AB-161 for indications of peripheral neuropathy, preclinical tolerability studies were conducted using rats and dogs, administering vehicle or AB-161 for 13 weeks with a 28-day recovery period.

In rats, AB-161 was tested at doses of 30, 120, and 500 mg/kg/day. Neurobehavioral and motor activity assessments were performed at pretreatment, during the main study (day 22), at the end of dosing (day 89), and at the end of recovery (day 119). When compared to vehicle controls, there were no test-article-related clinical observations or effects on body weight, food consumption, neurobehavioral assessments, or motor activity. There were no AB-161-related macroscopic/microscopic findings or organ weight changes at the end of the dosing or recovery periods, including no effects on liver, peripheral nerves, or male reproductive organs in rats.

In dogs, when tested at AB-161 doses of 10, 30, or 100 mg/kg/day, there were no AB-161-related effects on food consumption, electrocardiography (quantitative and qualitative), neurological examinations, and urinalysis and no ophthalmic or macroscopic findings.

**Figure 5:**
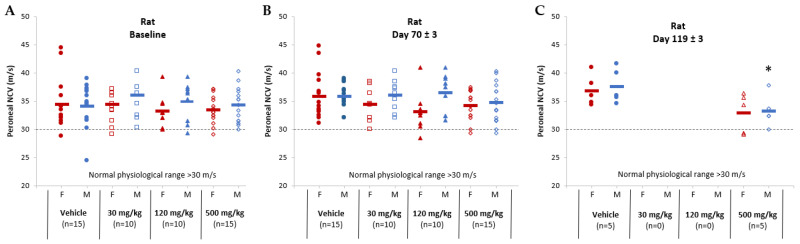
Nerve conduction velocity for the peroneal nerve among rats administered either vehicle or AB-161 (30 mg/kg, 120 mg/kg, 500 mg/kg). Individual female (red) and male (blue) rats were monitored for nerve conduction velocity (m/s) at (**A**) baseline, (**B**) near end of treatment (day 70 ± 3), and (**C**) end of recovery (day 119 ± 3). Normal physiological range (dotted reference line) for rat is >30 m/s. Mean for each group was determined and expressed as a horizontal line. Number of animals (n) are indicated underneath each group. * Indicates statistically significant difference (*p* < 0.05).

**Figure 6:**
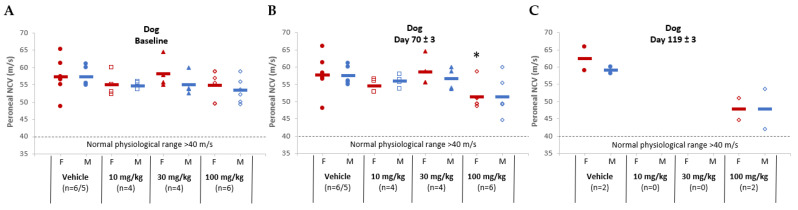
Nerve conduction velocity for the peroneal nerve among dogs administered either vehicle or AB-161 (10 mg/kg, 30 mg/kg, 100 mg/kg). Individual female (red) and male (blue) dogs were monitored for nerve conduction velocity (m/s) at (**A**) baseline, (**B**) near end of treatment (day 70 ± 3), and (**C**) end of recovery (day 119 ± 3). Normal physiological range (dotted reference line) for dog is >40 m/s. Mean for each group was determined and expressed as a horizontal line. Number of animals (n) are indicated underneath each group. * Indicates statistically significant difference (*p* < 0.05).

Nerve conduction velocity (NCV) results in both rats and dogs showed minor AB-161-related non-adverse NCV delays. In rats, NCV delays were noted in the high-dose groups (500 mg/kg) on day 70 and on day 119 (Figure 5). In dogs, peroneal sensory NCV delays were noted in 4 of 12 animals on days 71 to 72 from the high-dose group (100 mg/kg/day), which persisted at the day 119 timepoint in 1 male and 1 female (2 of 4 animals) from the recovery cohort (Figure 6). No differences from baseline or time-matched controls were noted for motor nerves in both rats and dogs. The observed NCV delays in the high-dose groups were considered non-adverse as all nerve conduction metrics remained within the physiologically normal range, with no coordination, behavioral, or histopathological correlates. However, AB-161-related degeneration/atrophy in the testis was noted in the 30 and 100 mg/kg/day group male dogs at terminal euthanasia and persisted at the end of recovery period at 100 mg/kg/day (Table 4 and Table 5). This observation was characterized by effects on the germinal epithelium that included degeneration, tubular depletion, vacuolation, disorganization, exfoliation, multinucleated germ cells, and individual cell necrosis. This finding correlated with the secondary effects in the epididymis consisting of cellular debris and decreased sperm cellularity in the 30 and 100 mg/kg/day group males at terminal euthanasia, which persisted at the end of the 1 month recovery period (100 mg/kg).

## 4. Discussion

HBsAg seroclearance in CHB has been reported to be associated with a reduced risk of adverse long-term clinical outcomes including liver decompensation and HCC [38]. Strategies through siRNA and ASO approaches to directly target HBV RNA for degradation by host RNA silencing machinery have shown promise in reducing HBsAg [39]. Notably, persistent suppression of HBsAg by imdusiran (AB-729), an HBV-targeting GalNAc-conjugated siRNA, was associated with the activation of HBV-specific T cells and the reduction of exhausted CD8+ T cells in some patients [40]. Sustained low levels of both HBV DNA and HBsAg following treatment discontinuation have been observed in some of the patients treated with imdusiran, suggesting that prolonged HBsAg suppression may restore immune control [41]. However, plateauing of HBsAg response occurred despite continued administration of siRNA [42], suggesting that combination treatment with other modulators will be needed to achieve a functional cure. In addition, siRNA and ASO therapeutics are administered by subcutaneous injection that is less convenient than oral dosing. Consequently, the development of agents that can reduce HBsAg and be delivered in oral dosage form would be highly desirable.

AB-161 is a pan-genotypic small-molecule HBV RNA destabilizer with potent in vitro and in vivo antiviral activities. HBV RNA destabilizers represent a class of orally bioavailable compounds with a differentiated mechanism of action compared to NA and CAM. These inhibitors target PAPD5/7, components of a virus–host interaction pathway that HBV employs to stabilize its transcripts [14]. Mechanistic studies using PAPD5/7 single and double knock-out in vitro systems revealed that PAPD5/7 plays a role in protecting viral RNA by preserving the integrity of its poly(A) tail [15]. Consistent with this mechanism, AB-161 inhibits the enzymatic activities of PAPD5/7, which leads to shortening and degradation of pgRNA and preS/S RNA, as indicated in the kinetic analysis of intracellular HBV RNA from cells treated with AB-161. Degradation of HBV pgRNA and preS/S RNA following treatment with AB-161 subsequently resulted in reduction of HBV DNA and viral proteins including HBV core, HBeAg, and HBsAg. Genetic studies also verified that the activity of AB-161 was dependent on the presence of the SLα sequence within the HBV PRE, which plays a role in recruiting PAPD5/7 onto HBV RNAs [14]. Since AB-161 inhibits HBsAg produced from both cccDNA and the partially integrated HBV transcripts, the data suggest that the SLα sequence is retained upon integration. However, since only the PLC/PRF/5 cells were used as a cell-based model for integrated HBV, the antiviral effect of AB-161 against transcripts from the integrated HBV genome would need to be further investigated. As integrated HBV transcripts represent the dominant source of HBsAg in HBeAg negative patients, an effective antiviral that aims to reduce HBsAg should target transcripts from both cccDNA and integrated HBV. In addition, AB-161 retains activity against NA-resistant variants and shows additivity with NA and CAM, supporting the evaluation of AB-161 in future combination studies with other classes of HBV inhibitors.

Among first-generation HBV RNA destabilizers, many have been discontinued from further development, where the major safety findings appeared to be associated with neurotoxicity during preclinical studies [23,24,25]. In the case of GS-8873, 13-week dosing led to significant reductions in nerve conduction velocity across all dose groups in both rats (2, 6, 20, or 60 mg/kg/day) and monkeys (0.5, 1.5, 3, or 6 mg/kg/day), along with axonal degeneration in rat spinal and peripheral nerves compared to vehicle controls [24]. Similarly, long-term dosing of RG7834 in rats (6 months) and monkeys (9 months) also identified dose- and time-dependent neurotoxicity, along with axonal degeneration in peripheral nerves and spinal cord across all dose groups [23]. Our first-generation compound, AB-452, was also discontinued due to observations of neurobehavioral changes in dogs, along with axonal degeneration of sciatic and spinal nerves in rats and dogs, respectively. To mitigate neurotoxicity for our second-generation compound, our strategy involved developing a molecule with a liver-centric profile. We demonstrated in vivo proof of concept for the liver-centricity of AB-161 using the AAV-HBV mouse model, in which suppression of HBsAg was driven by high liver concentrations. In particular, a dose-dependent effect in HBsAg reduction was observed when the unbound fraction of AB-161 in liver was higher than the EC_90_ when compared to the levels in plasma, which were substantially lower than the EC_90_ across all doses. Furthermore, plasma protein binding of AB-161 was substantially higher (95% in human plasma) compared to first-generation HBV RNA destabilizers; protein binding was about 40% for GS-8873, 50% for AB-452, 56% for RG7834, and 57% for GST-HG131 [23,24,25]. High plasma protein binding can impact free compound availability and potentially reduce toxicity due to lower systemic exposures. 

The 13-week toxicology studies in rats and dogs were performed based on our prior experience with AB-452, in which dogs appeared to be the more sensitive species, as long-term dosing resulted in neurobehavioral changes and histopathology findings in nerve tissues. With AB-161, contrary to AB-452, there were no NCV changes noted for motor nerves in all dose groups, and only non-adverse sensory NCV delays were observed with the high-dose group at day 70 ± 3 and day 119 ± 3 post-dose (end of recovery) during preclinical 13-week toxicology studies. All nerve conduction metrics remained within physiologically normal ranges in the affected animals, and there were no neurobehavioral or histopathology correlates based on nervous system tissues assessments in both rats and dogs. Although the risk of neurotoxicity may be associated with increased exposure, these data suggest that the liver-centric and high protein-binding profile of AB-161 appeared to have improved the neurotoxicity safety concern with respect to first-generation HBV RNA destabilizers. 

Despite our effort to mitigate the neurotoxicity risk, test-article-related microscopic findings were observed in the testis and epididymis of male dogs. There were no reproductive safety findings identified in female dogs or in male or female rats during the 13-week administration across all doses. The mechanism of AB-161-induced male reproductive toxicity in dogs is unknown. Similar adverse events associated with abnormal sperm and testicular changes in rats have previously been reported for RG7834 [23,43]. Decreased epididymis and minimal-to-mild spermatid depletion were also observed with prolonged dosing of AB-452 in rat male reproductive tissues. Clinically, male reproductive parameters (e.g., semen analysis, sperm DNA fragmentation, and sex hormone levels) were monitored during single and multiple ascending dose evaluations of GST-HG131 [22]. While there were no clinically significant findings, the monitoring of male reproductive parameters in Phase 1 studies suggests that adverse findings were likely observed during preclinical toxicology assessments of GST-HG131. Although an off-target effect may be a possibility since male reproductive toxicity was not reported for GS-8873, these adverse concerns with respect to the aforementioned HBV RNA destabilizers suggest that inhibiting PAPD5/7 could be a possible mechanism of toxicity as these compounds share the same pharmacological targets. Based on these on/off-target adverse toxicities, targeting PAPD5/7 has proven to be extremely challenging and more in-depth studies are required to elucidate a mechanism for these toxicities. 

In conclusion, we discovered a next-generation HBV RNA destabilizer, which showed potent anti-HBV activities in vitro and inhibited HBsAg production in vivo using AAV-HBV transduced mice. Analysis of plasma and liver exposures demonstrated that the antiviral effect was driven by high liver concentrations of AB-161. Compared to our first-generation compound (AB-452), AB-161 showed improvements in neurotoxicity profile in preclinical 90-day toxicology studies. Unfortunately, adverse findings associated with male reproductive toxicity were observed during the 90-day toxicology studies in dogs. Considering the high bar for safety expected for novel CHB treatment and the complexity of monitoring reproductive toxicity, development of AB-161 was not supported and was discontinued from further progression.

## Figures and Tables

**Table 1:** AB-161 suppresses HBV antigen and viral replication in HBV cell-based systems.

HBV System	Genotype	HBV Markers	EC_50_ [nM]	CC_50_ [μM]
HepG2.2.15	D	HBsAg HBsAg (40% human serum) HBeAg HBV DNA	2.3 ± 1.0 63 ± 29 13.7 ± 1.7 0.36 ± 0.2	>30
HBV-infected PHH	D	HBsAg HBeAg HBV RNA	8.5 ± 4.7 11.9 ± 2.5 9.6 ± 9.1	>10
HBV-infected HepG2-hNTCP	D	HBsAg HBeAg HBV RNA	25.3 ± 10.9 20.9 ± 5.0 30.1 ± 9.6	>10
PLC/PRF/5	D	HBsAg	5.9 ± 2.5	>10
HBV transfected Huh-7	A–H	HBsAg	2.1 to 4.5	>50

Data represent average values and standard deviations from at least three independent experiments.

**Table 2:** HBV variants resistant to nucleos/(t)ide analogs remained susceptible to AB-161.

HBV Polymerase Variant	ETV HBV DNA, EC_50_ [μM]	GLS4 HBV DNA, EC_50_ [μM]	AB-161 HBsAg, EC_50_ [μM]
Wild-type	0.017	0.075	0.0049
rtL180M/M204V	0.28	0.040	0.0019
rtL180M/M204V/T184G/S202I	>6.3	0.084	0.0020

**Table 3:** In vitro combination effects on HBV DNA of AB-161 with ETV, TDF, or AB-836 using HepG2.2.15 cells.

Inhibitor A	Inhibitor A EC50 (µM) ^a^	AB-161 EC50 (µM) ^a^	Synergy (µM^2^%) ^b^	Antagonism (µM^2^%) ^b^	Conclusion ^b^
ETV	0.0051 ± 0.0011	0.0027 ± 0.0008	0.00 ± 0.00	−3.99 ± 3.59	Additive
TDF	0.0136 ± 0.0044	0.0019 ± 0.0006	0.00 ± 0.00	−0.43 ± 0.744	Additive
AB-836	0.0054 ± 0.0007	0.0021 ± 0.0009	0.47 ± 0.82	−9.61 ± 6.71	Additive

^a^ EC_50_ values indicate mean ± standard deviation from three independent experiments. ^b^ Prichard and Shipman interpretive criteria at 99.9% confidence: Synergy or antagonism volumes (µM^2^%) <25 = probably insignificant; 25–50 = minor but significant, 50–100 = moderate; >100 = strong synergy or strong antagonism.

**Table 4:** Summary of testis weight data after terminal euthanasia at the end of the main study (days 97–100) and after a 28-day recovery period (Day 120) in the 13-week dog tolerability studies.

	Males			
Group Dose (mg/kg/day) No. animals per group (main study) No. animals per group (recovery)	1 0 3 ^a^ 2	2 10 4 0	3 30 3 ^b^ 0	4 100 4 2
End of main study: Testis (No. weighed) Absolute value ^c^ % of brain weight ^c^ % of body weight ^c^	3 14.77 g 19.96 0.15	4 −18.03 −16.13 −9.38	3 −34.17 −30.73 −21.93	4 −20.62 −16.10 −12.18
End of recovery: Testis (No. weighed) Absolute value ^c^ % of brain weight ^c^ % of body weight ^c^	(2) 15.43 g 20.18 0.16	NA	NA	(2) −42.84 −43.49 −37.79

NA = not applicable; g = grams. ^a^ Single animal euthanized on day 41 due to reasons unrelated to test article. ^b^ Single animal euthanized on day 77 due to reasons unrelated to test article. ^c^ Control group expressed as mean values; all other values expressed as percent difference from the control group means.

**Table 5:** Incidence of AB-161 histopathology findings at the end of main study (day 97–100) and after 28-day recovery period (Day 120) in the 13-week dog tolerability studies.

	Males			
Group Dose (mg/kg/day) No. of animals per Group (main study) No. animals per group (recovery)	1 0 3 2	2 10 4 0	3 30 3 0	4 100 4 2
End of main study: Testis (no. examined) Degeneration/Atrophy Minimal Mild Moderate	(3) 0 - - -	(4) 0 - - -	(3) 2 1 1 0	(4) 4 2 1 1
End of recovery: Testis (no. examined) Degeneration/Atrophy Moderate	(2) 0 -	NA	NA	(2) 2 2
End of main study: Epididymis (no. examined) Cellular debris Minimal Mild Cellularity, decreased; sperm Minimal Mild Marked	(3) 0 - - 0 - - -	(4) 0 - - 0 - - -	(3) 2 0 2 2 0 2 0	(4) 4 2 2 4 1 2 1
End of recovery: Epididymis (no. examined) Cellular debris Mild Cellularity, decreased; sperm Moderate	(2) 0 - 0 -	NA	NA	(2) 2 2 2 2

NA = not applicable; - = no AB-161-related findings.

## Data Availability

Data presented in the study are included in the article and supplementary materials.

## Supplementary Materials

The following supporting information can be downloaded at https://www.mdpi.com/article/10.3390/v16030323/s1: Table S1: HBV genotype coverage of AB-161; Table S2: Antiviral selectivity and cytotoxicity of AB-161; Figure S1: Stem-loop alpha sequence within the HBV PRE is critical for AB-161 antiviral activity; Figure S2: AB-161 once-daily dosing is well-tolerated in AAV-HBV mice.
