# Supplementary material for: Preclinical Antiviral and Safety Profiling of the HBV RNA Destabilizer AB-161
Source: Viruses. 2024 Feb 21;16(3):323. doi: 10.3390/v16030323 (PMC10975527; doi:10.3390/v16030323)
Supplement: Supplementary file 1 [file viruses-16-00323-s001.zip › viruses-2870925-supplementary.pdf]

Supplementary Materials for

Preclinical Antiviral and Safety Profiling of the HBV RNA Destabilizer AB-161

Angela M Lam*, Ravi R Dugyala, Muhammed Sheraz, Fei Liu, Emily P Thi, Ingrid Graves, Andrea Cuconati, Holly Micolochick Steuer, Andrzej Ardzinski, Nathan Overholt, Jeremy D Mason, Dimitri Gotchev, Andrew G Cole, Troy O Harasym, and Michael J Sofia*

Arbutus Biopharma, Inc., 701 Veterans Circle, Warminster, PA 18974
*Correspondence: alam@arbutusbio.com (A.M.L.); msofia@arbutusbio.com (M.J.S.)

Table S1. HBV genotype coverage of AB-161.

HBV genotype	HBsAg EC_{50} [nM]	CC_{50} [μM]
A	3.0 ± 0.8	>50
B	2.6 ± 1.4	>50
C	2.3 ± 0.6	>50
D	4.5 ± 2.8	>50
E	2.5 ± 0.9	>50
F	3.3 ± 2.6	>50
H-1	2.4 ± 0.5	>50
H-2	2.1 ± 0.9	>50

Data represent average value and standard deviations from at least 3 independent experiments.

Table S2. Antiviral selectivity and cytotoxicity of AB-161.

Virus[1]	Family (genome)	Cell line (tissue origin)	EC_{50} [μM]	CC_{50} [μM]
HBV	*Hepadnaviridae* (dsDNA)	HepG2.2.15 (human liver)	0.0023	>30
HCV	*Flaviviridae* (+ssRNA)	Huh-7 (human liver)	>30	>30
DENV2	*Flaviviridae* (+ssRNA)	BHK21 (hamster kidney)	>30	>30
WNV	*Flaviviridae* (+ssRNA)	Vero76 (monkey kidney)	>30	>30
HCMV	*Herpesviridae* (dsDNA)	MRC-5 (human lung)	>30	>30
HSV-1/HSV-2	*Herpesviridae* (dsDNA)	MRC-5 (human lung)	>30	>30
IAV	*Orthomyxoviridae* (-ssRNA)	MDBK (bovine kidney)	>30	>30
RSV	*Paramyxoviridae* (-ssRNA)	HEp2 (human epithelial)	>30	>30
HRV	*Picornaviridae* (+ssRNA)	HEp2 (human epithelial)	>30	>30
HIV	*Retroviridae* (+ssRNA)	CEM-22 (human T lymphoblast)	>30	>30

Abbreviations: HBV = hepatitis B virus; HCV= hepatitis C virus; DENV2 = dengue virus type 2; WNV = West Nile virus; HCMV = human cytomegalovirus; HSV-1/HSV-2 = herpes simplex virus-1/herpes simplex virus-2; IAV = influenza A virus; HRV = human rhinovirus; RSV = respiratory syncytial virus; HIV = human immunodeficiency virus.

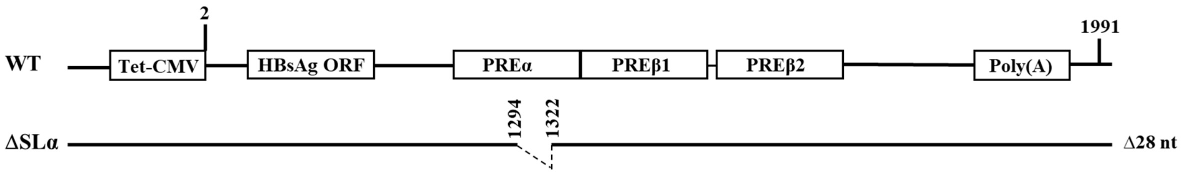

Plasmid	AB-161 HBsAg EC_{50} [nM]
WT	2.5 ± 2.6
ΔSLα	>100

Figure S1. Stem-loop alpha sequence within the HBV PRE is critical for AB-161 antiviral activity. **(Top)** Schematic representations of plasmids encoding the open reading frame of HBsAg and either wild type (WT) or SLα deleted (ΔSLα) PRE sequence. **(Bottom)** Huh-7 cells transfected with WT or ΔSLα plasmids were treated with varying concentrations of AB-161. Levels of HBsAg were monitored to determine the effect of AB-161 against WT or the stem-loop alpha deletion mutant.

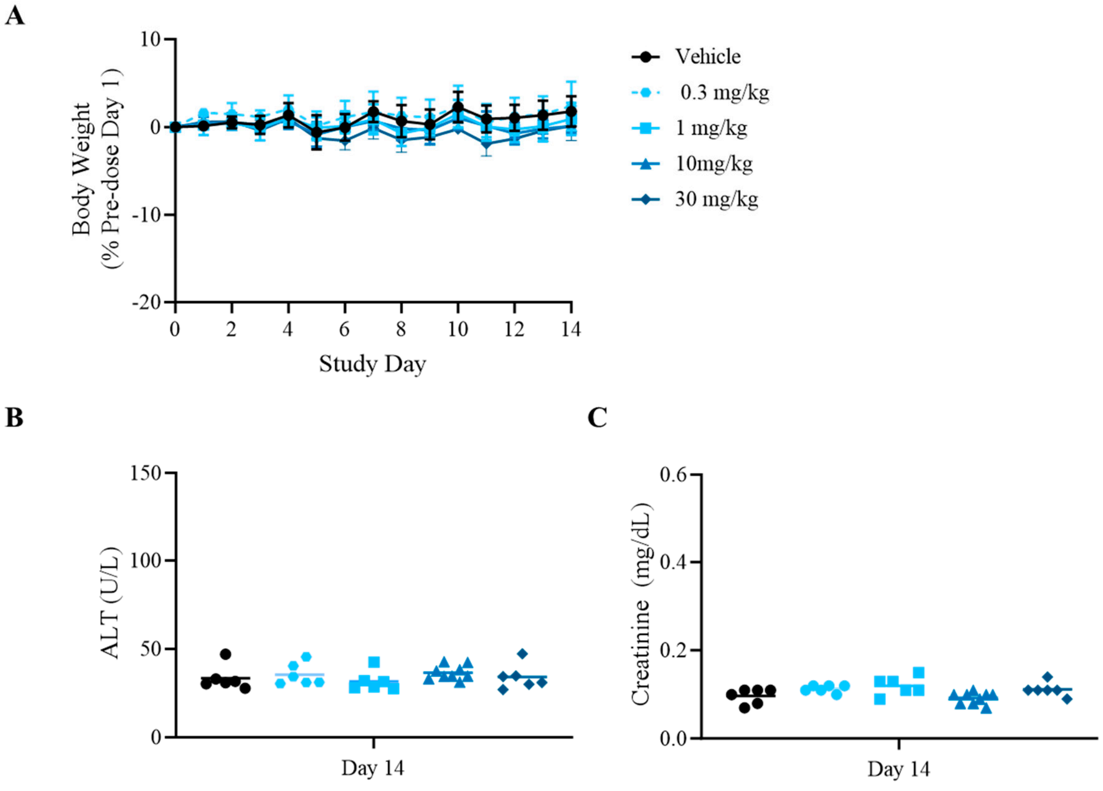

Figure S2. AB-161 once-daily dosing is well-tolerated in AAV-HBV mice. **(A)** AAV-HBV mice received once-daily oral doses of AB-161 or vehicle control from Days 0 to 13. Data represent body weights relative to pre-dose baseline levels (n = 6–9) of group mean ± standard deviation. **(B)** Alanine aminotransferase (ALT) and **(C)** creatinine levels were measured in serum as markers of liver and kidney function, respectively. Data represent group mean (n = 6–9) ± standard deviation.
